# Identification and Functional Analysis of Interleukin-1β in the Chinese Soft-Shelled Turtle *Pelodiscus sinensis*

**DOI:** 10.3390/genes7050018

**Published:** 2016-05-04

**Authors:** Quan Liang, Weifen Li, Ningning Guo, Chao Tong, Yingshan Zhou, Weihuan Fang, Xiaoliang Li

**Affiliations:** 1 Key Laboratory of Molecular Animal Nutrition, Ministry of Education, College of Animal Science, Zhejiang University, Hangzhou 310058, China; 10917014@zju.edu.cn (Q.L.); wfli@zju.edu.cn (W.L.); 2 Zhejiang Provincial Key Laboratory of Preventive Veterinary Medicine, Institute of Preventive Veterinary Medicine, Zhejiang University, Hangzhou 310058, China; pitt_lq@foxmail.com (N.G.); tongchao00@gmail.com (C.T.); yszhou@zju.edu.cn (Y.Z.); whfang@zju.edu.cn (W.F.)

**Keywords:** *Pelodiscus sinensis*, Interleukin-1β, Caspase-1, ICE cut site

## Abstract

Chinese soft-shelled turtle (*Pelodiscus sinensis*) is commercially cultured in East and Southeast Asia for its nutritional and medicinal values. In this study, we identified interleukin-1β (IL-1β) from Chinese soft-shelled turtle. The full-length cDNA of *Pelodiscus sinensis* IL-1β (tIL-1β) consists of 1529 base pairs with an 831-base-pair open reading frame, encoding 277 amino acids. The guanine-cytosine (GC) content in the coding sequence and 3’ untranslated region of tIL-1β is considerably higher than that of other vertebrates. Its mRNA expression level increased significantly during *Aeromonas hydrophila* infection. The tIL-1β lacks the typical IL-1β-converting enzyme (ICE) cut site found in mammalian IL-1β, but still could be cleaved by turtle caspase-1. By mutating the potential cleavage sites, we identified aspartic acid (Asp/D) 130 as the ICE cut site in tIL-1β. The peptide truncated at D130 was expressed using the baculovirus expression system; its bioactivity is confirmed by the ability to induce cyclooxygenase-2 (COX-2) and tIL-1β itself in peripheral blood monocytes. In conclusion, we characterized IL-1β from Chinese soft-shelled turtle and identified its D130 as a non-typical ICE cut size.

## 1. Introduction

Interleukin-1β (IL-1β), belonging to the interleukin-1 (IL-1) cytokine family, is a prototypical proinflammatory cytokine and key mediator of the body’s response to microbial infection, immunological reactions and tissue injury [1,2]. It is produced as an inactive precursor that has to be cleaved to form a biologically active mature peptide. Different proteases are capable of processing IL-1β precursor to active form. Caspase-1 is the first identified and the most important protease involved in IL-1β processing [3,4]. Other proteases such as granzyme A [5], elastase, trypsin and chymotrypsin [6] have also demonstrated the ability of processing IL-1β precursor extracellularly. IL-1β is mainly secreted by blood monocytes, tissue macrophages and dendritic cells [7]. It does not follow the classical endoplasmic reticulum-to-Golgi pathway of secretion due to lack of the signal peptide [8]. Extracellular ATP acting via the P2X7 receptor to induce caspase-1-dependent release of IL-1β is the best-known secretion mechanism [7]. Extracellular mature IL-1β peptide binds to IL-1 receptor 1 (IL-1R1), leading to the recruitment of IL-1 receptor accessory protein (IL-1RAP) with the formation of a heterodimer complex that activates the nuclear factor-κB (NF-κB) and mitogen-activated protein kinase (MAPK) pathways in a MyD88-dependent pattern [9,10]. This result is in the upregulation of several inflammatory genes such as IL-1β, tumor necrosis factor-α (TNF-α) and cyclooxygenase-2 (COX-2) [11]. Because of its potency and extensive functions, the biological activity of IL-1β is tightly regulated. IL-1β is upregulated during inflammation or microbial infection and then declines rapidly, a process attributed to the presence of unstable elements in its mRNA [12,13], microRNA regulation and negative feedback mechanisms [14].

Genes encoding IL-1β have been characterized from a number of mammalian and non-mammalian vertebrates, including human [15], mouse [16], cow [17], sheep [18], pig [19], chicken [20], trout [21], seabass [22], clawed frog [23] and catshark [24], but not in reptilian species. These IL-1β sequences reveal that IL-1β of mammals contains a typical conserved interleukin-1β-converting enzyme (ICE) cut site which is absent in non-mammals [2]. The Chinese soft-shelled turtle, *Pelodiscus sinensis* (*P. sinensis*), is an ectothermic amniotic reptile and serves as an important evolutionary link between ectothermic anamniotic fish and amphibians, and endothermic amniotic birds and mammals [25]. The freshwater turtle is regarded as health-promoting food in Asian countries such as China, Japan and Korea. Emergence of a variety of infectious diseases is concurrent with the recent large-scale farming of Chinese soft-shelled turtle [26,27,28]. However, little information is available on the immunity of turtles or immune responses upon infection. In this study, we identified and characterized IL-1β gene from Chinese soft-shelled turtle and investigated its ICE cut site.

## 2. Materials and Methods

### 2.1. Preparation of Tissue cDNA Samples

Healthy Chinese soft-shelled turtles (~500 ± 30 g) were obtained from Hangzhou Zhongde Aquatic Culture Co., Ltd. (Hangzhou, China). Turtles were euthanized using diethyl ether and different tissues were collected and stored under liquid nitrogen until analysis. Total RNA extraction and cDNA synthesis were performed as previously described [10]. Briefly, total RNA was extracted from tissue samples using the RNAprep pure Tissue Kit (Tiangen, Beijing, China) and treated with DNase I (Takara, Dalian, China) at 37 °C for 30 min. Purity and quantity of RNA were measured on NanoDrop spectrophotometer (Thermo Scientific, Wilmington, DE, USA). Integrity of RNA was tested by agarose gel electrophoresis. Only intact RNA samples with 260:280 nm ratio between 2.0 and 2.2 were used for cDNA synthesis. All animal experiments in this study were approved by the Laboratory Animal Management Committee of Zhejiang University (Approval No. 2013120903).

### 2.2. Cloning of the Genes Encoding Turtle Interleukin-1β (IL-1β) and Caspase-1

A partial fragment of the tIL-1β gene was amplified using the degenerate primer pair tIL-1-dF and -dR (Table S1). Synthesized cDNA from spleen was used as a template in PCR reaction with a denaturation step of 95 °C for 3 min, followed by 40 cycles of 94 °C for 30 s, 50 °C for 30 s and 72 °C for 30 s, and completed with a 10 min extension at 72 °C.

The 5’ end RACE was performed using GeneRacer Kit (Invitrogen, Eugene, OR, USA) according to the manufacturer’s instructions. The cDNA fragments of tIL-1β were amplified by two rounds of PCR with gene-specific primers and adapter primers provided in the Kit (a set of GeneRacer 5’ Primer and tIL-1-R1 for the first round, and GeneRacer 5’ nested primer and tIL-1-R2 for the second round PCR). The 3’ end RACE was also performed with the GeneRacer Kit. The gene-specific primers, the tIL-1-F1 and tIL-1-F2, were designed based on the tIL-1β partial fragment. The primer pairs, tIL-1-F1 and GeneRacer 3’ primer, and tIL-1-F2 and GeneRacer 3’ nested primer were used for the first and second round of PCR, respectively.

The primer sets for turtle caspase-1 (tcaspase-1) were designed using our preliminary transcriptome data of turtles infected with *A. hydrophila*. The sequence of chicken caspase-1 was used to search the turtle caspase-1 transcript with the Blast program. The complete tcaspase-1 ORF were amplified with primer sets tcas1-ORF-F and -R. The gene fragments were then cloned into pMD-18T (Takara) and sequenced for analysis.

To examine the gene organization of intron and exon in tIL-1β gene, genomic DNA of the Chinese soft-shelled turtle was extracted from blood sample using the animal genomic DNA extraction Kit (Tiangen). The tIL-1β gene was amplified with nested PCR using the primer sets tIL-1-g1F and -g1R for the first round and tIL-1-g2F and -g2R for the second round. PCR product was cloned into pEASY-blunt (TransGen, Beijing, China) and sequenced. The gene organization was determined with the SIM4 program [29].

### 2.3. Sequence Analysis

The full-length cDNA of tIL-1β and tCaspase-1 ORF were analyzed using Blastn programs (http://www.ncbi.nlm.nih.gov/BLAST/). Predicted amino acid sequence was used in searching homology domains in the Pfam database (http://pfam.sanger.ac.uk/search). The secondary structure of the tIL-1β protein was predicted using PSIPRED v3.0 (http://bioinf.cs.ucl.ac.uk/psipred/). Multiple sequence alignment was carried out using the CLUSTALW2.1 program (http://www.genome.jp/tools/clustalw/). The phylogenetic tree was constructed with the MegaV5.0 software package (http://www.megasoftware.net/) using the neighbor-joining method and bootstrapped 10,000 times.

### 2.4. Preparation of Polyclonal Antibodies Against tIL-1β

Truncated tIL-1β sequence-encoding amino acids 118–276 (predicted as 19 kDa mature peptide) were amplified using the primer set rtIL-1-F and -R. The fragment was then cloned into pET32a(+) and verified by sequencing. The recombinant tIL-1β was expressed using the prokaryotic expression system and purified with Ni-IDA agarose (Weisi Co. Ltd., Beijing, China) as previously described [30]. The size and purity of the protein was confirmed by SDS-PAGE and Western blotting using anti-His(6)-Tag antibody (Sigma, St. Louis, MO, USA). Protein concentration was determined with the bicinchoninic acid (BCA) assay kit (Thermo Scientific).The New Zealand white rabbits (3–4 kg body weight) were used for the production of polyclonal antibodies. tIL-1β protein (100 μg) in 500 μL of sterile 10 mM phosphate-buffed saline (PBS) was emulsified with 500 μL complete Freund’s adjuvant and injected into muscle at multiple sites. Subsequent immunizations were given with incomplete Freund’s adjuvant. After three immunizations at a two-week interval followed by a booster after six weeks, the rabbits were bled at weekly intervals and the titer was determined by indirect ELISA. Collected antibody was then used for Western blotting at a dilution of 1:500 as described below.

### 2.5. Quantitative PCR

Quantitative PCR (qPCR) was carried out to examine relative transcription levels of target genes using β-actin as the internal reference. Fluorescence signals from iTaq™ Universal SYBR® Green Supermix (Bio-Rad, Hercules, CA, USA) were captured on the iQ5™ real-time multicolor PCR detector (Bio-Rad). The cDNAs were synthesized from total RNA using the Oligo(dT) 18 Primer (Takara). The primer sets used in qPCR are listed in Table S1. All qPCR primer sets were verified as having amplification efficiency of 95%–105% and their amplification specificities were confirmed by analyzing the melting curve as a single peak with each primer set. The reaction conditions were slightly adjusted as previously described [30]. Fold changes of the gene transcripts were calculated as 2^-^^ΔΔCt^ [31].

### 2.6. tIL-1β Expression Analysis in Healthy or Aeromonas Hydrophila-Infected Turtles

Liver, lung, small intestine, kidney, heart and peripheral whole blood were collected from five turtles. All tissues were frozen immediately in liquid nitrogen until extraction of total RNA. qPCR was performed as described above, β-actin was used as internal control and the results were presented as the relative level to kidney.

There were 24 turtles (400–500 g) acclimated in individual tanks for 7 days. Four turtles were then euthanized to collect liver, kidney, small intestine, spleen and blood samples as day 0 controls. The remaining turtles were intraperitoneally infected with 1 mL of suspension containing about 1 × 10^8^ CFU of *A. hydrophila* strain T6. Four turtles were euthanized to collect the tissue samples each time at 6, 12, 24, 48 and 72 h post-infection. Total RNA was extracted for qPCR analysis of tIL-1β mRNA expression, which was shown as fold changes relative to the day 0 controls (*i.e.*, uninfected turtle tissue). Proteins in the spleen samples were extracted as previously described [30].

### 2.7. Plasmid Construction and Transfection

ORF of tIL-1β was amplified using primers tIL-1-Flag-F and -R and cloned into pcDNA3.1-3’Flag to construct pcDNA3.1-tIL-1β-Flag. ORF of tcaspase-1 with C-terminal HA tag was amplified using primers tCasp1-HA-F and -R and cloned into pcDNA3.1(+) to generate pcDNA3.1-tcsap1-HA. The recombinant plasmid was confirmed by sequencing. Recombinant plasmids were extracted with EndoFree Plasmid Extraction Kit (Omega, Norcross, GA, USA) and concentrations were measured with NanoDrop (Thermo Scientific). HEK293 cells were transiently transfected with pcDNA3.1-tIL-1β-Flag and/or pcDNA3.1-tcsap1-HA using Lipofectamine 2000 (Invitrogen), pcDNA3.1-3’Flag and/or pcDNA3.1(+) were used as controls. Cells pellet fractions were lysed 36 h post-transfection with IP/Western blot Lysis buffer (Beyotime, Haimen, China) containing protease inhibitor cocktail (Roche, Basel, Switzerland). For the detection of secreted tIL-1β, cell supernatant was collected and centrifuged at 1500 ×*g*, 4 °C for 5 min to remove the suspended cells. Cell-free supernatant was immunoprecipitated with anti-tIL-1β polyclonal antibody using Crosslink Magnetic IP and Co-IP Kit (Thermo Scientific). Lysed cell pellet and immunoprecipitated supernatant were harvested for Western blotting.

The amino acid substitutions of D89A, D91A, D97A, D130A, and D132A were introduced into pcDNA3.1-tIL-1β-Flag with QuickChange Site-Directed Mutagenesis Kit (Stratagene, La Jolla, CA, USA) and verified by sequencing. pcDNA3.1-tIL-1β-Flag/mutated plasmids and pcDNA3.1-tcasp1-HA were delivered into HEK293 cells by transfection with lipofectamine 2000 (Invitrogen). Cells were collected and Western blotting was used to detect the tIL-1β polypeptide as described below.

### 2.8. Expression of Turtle IL-1β in the Baculovirus Expression System

pFastBacHT-melittin used in the following experiments was derived from pFastBacHT (Invitrogen); it is modified by insertion of sequence-encoding melittin between polyhedrin promoter and multiple cloning site. Full-length of tIL-1β ORF or truncated tIL-1β (aa 131–276, about 17 kDa), amplified with primers tIL-1β-Bac-F and -R or primers tIL-1β-Bac-131-F and -R, both containing C-terminal His tag, was inserted into pFastBacHT-melittin vector by using In-Fusion cloning kit (Clontech, Palo Alto, CA, USA). The plasmids were verified by sequencing and transformed into DH10Bac (Invitrogen). Bacmid of verified colony was extracted and transfected into Sf9 cells using Cellfectin II Reagent (Invitrogen). The first passage recombinant baculovirus stocks (P1) were collected at 5 days post-transfection and used to infect the insect cells to propagate the recombinant viruses at 27 °C. Titers of the P2 baculovirus stocks were determined and used to infect the cells cultured in serum-free medium Sf900 (Invitrogen). After 72 h of incubation, the cultures were centrifuged at 8000 ×*g* for 10 min, and the supernatant samples were dialyzed in 10 mM PBS containing 10% glycerol for 24 h. Secreted proteins were purified using Ni-IDA agarose (Weisi, Beijing, China). The tIL-1β proteins were buffer exchanged with PBS using the Amicon^®^ Ultra-3kDa (Millipore, Bedford, MA, USA) and concentration was determined with BCA kit (Thermo Scientific). The final concentration was adjusted to 200 µg/mL.

### 2.9. Transcriptional Analysis of Inflammatory Cytokines in Turtle Peripheral Blood Monocytes Stimulated with tIL-1β

For isolation of peripheral blood monocytes (PBMo), blood samples from the jugular vein of healthy turtles were collected into a 50 mL centrifuge tube pre-filled with 200 µL of 1% heparin sodium (Sangon, Beijing, China). Histopaque 1083 (Sigma) was used for gradient separation according to the manufacturer’s instruction. Isolated PBMo was washed twice with RPMI-1640 (Gibco, Grand Island, NY, USA) and resuspended in complete RPMI-1640 medium supplemented with 10% heat-inactivated newborn calf serum (Gibco), 100 units/mL penicillin and 10 µg/mL streptomycin. PBMo was cultured in 24-well plates for 12 h to allow adhesion. The adherent cells were washed once with RPMI-1640, and incubated in complete RPMI-1640 medium containing tIL-1β (expressed in the baculovirus system) at the final concentration of 200 ng/mL. Lipopolysaccharide (LPS, Sigma) was used at a final concentration of 1 μg/mL as positive control and RPMI-1640 medium was used as negative control. The mRNA transcription levels of *cox-2, il-18, il-1β, casp-1* and *β-actin* were determined by qPCR after stimulation with tIL-1β peptide or LPS or RPMI-1640 at 1, 2 and 4 h post-treatment, respectively. Each treatment consisted of three replicates.

### 2.10. Western Blotting

Western blotting was conducted as previously described [30]. The protein samples were separated on 12% SDS-PAGE and transferred onto PVDF membrane. The membrane was blocked with 5% non-fat milk, incubated with the primary antibody and peroxidase conjugated goat anti-rabbit IgG as a secondary antibody. The blots were revealed on the ChampChemi^TM^ professional imaging system (SageCreation Science, Beijing, China) in the presence of Supersignal^®^ west pico-chemiluminescent substrate system (Thermo Scientific).

### 2.11. Statistical Analysis

All results in figures were presented, where appropriate, as means ± the standard deviations from three independent experiments and analyzed by using the Mann–Whitney U test (By GraphPad Prism software package, GraphPad Software, La Jolla, CA, USA). Statistical significance was determined at *p* < 0.05 (*) or *p* < 0.01 (**) level.

## 3. Results

### 3.1. Cloning of the Full-Length tIL-1β cDNA

**Figure 1:**
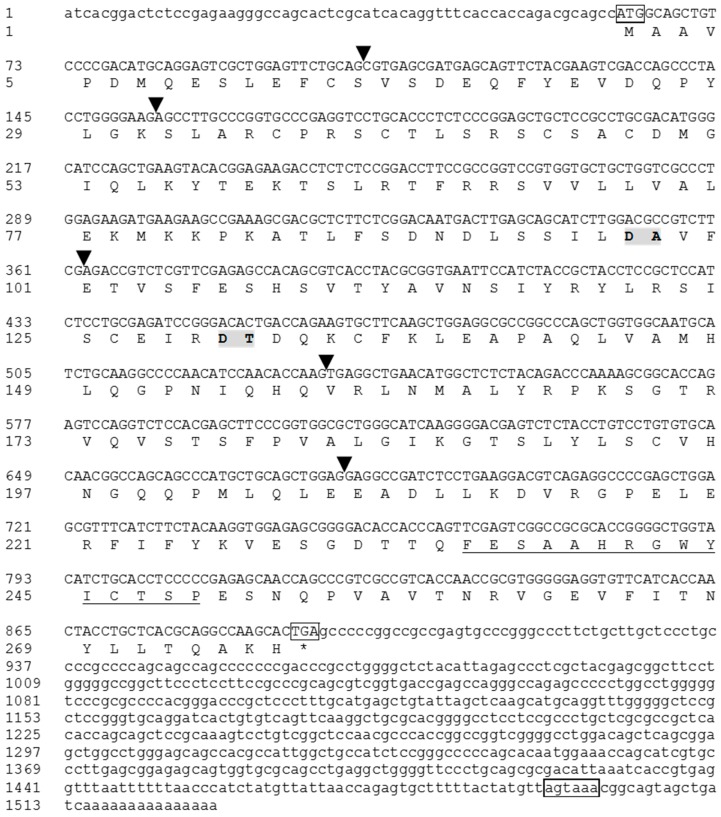
Nucleotide and amino acid sequence of *P. sinensis* IL-1β. The start and stop codons and polyadenylation signal (AGTAAA) in the 3’-UTR are boxed. The IL-1 family signature is underlined. Predicted IL-1-converting enzyme cut site is highlighted in grey. The intron position is indicated with a triangle.

**Figure 2:**
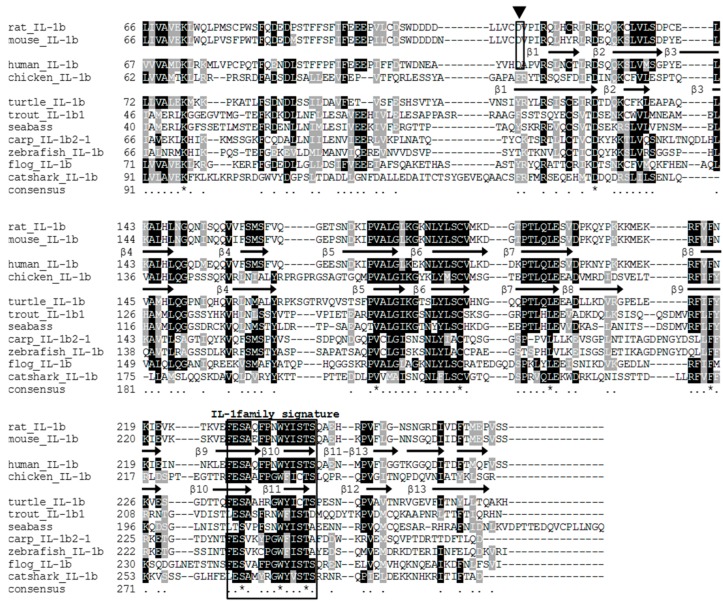
Multiple alignment of *P. sinensis* IL-1β molecules with known IL-1βs by ClustalW. Conserved amino acids are shaded using BOXSHADE. The IL-1 family signature is labeled above the alignment and the human IL-1β β-sheet and predicted *P. sinensis* IL-1β β-sheet is indicated with an arrow. Mammalian IL-1-converting enzyme cut site is labeled with triangle. The accession numbers of the IL-1β sequences are as follows: NP_113700.2 (rat), EDL28238 (mouse), NP_000567 (human), NP_989855 (chicken), AFX97767.1 (turtle), CAB53541.3 (trout), CAC41006.1 (seabass), CAC19887.1 (carp), AAQ16563.1 (zebrafish), CAC85480.1 (flog), CAC09435.1 (catshark).

The full-length cDNA of tIL-1β (GenBank Accession No. JX846915) contains a 61 bp 5’-untranslated region (UTR), a 831 bp ORF encoding 277 amino acids (aa) with a predicted molecular weight of 30 kDa, and a 637 bp 3’-untranslated region (Figure 1). A variant polyadenylation signal AGTAAA was found in the 3’ UTR (Figure 1). The predicted amino acids sequence contained the IL-1 family signature motif [FC]-x-S-[ASLV]-x(2)-P-x(2)-[FYLIV]-[LI]-[SCA]-T-x(7)-[LIVM] in the C-terminus [2]. An IL-1 family domain was also present in aa 121–272, as revealed by SMART program search. SignalP analysis showed that turtle IL-1β lacks the N-terminus signal peptide. Secondary structure analysis identified twelve β-sheets in the C-terminus (Figure 2). BLAST search with the ProDom database showed the presence of a domain similar to PDA5A3H2 (domestic pigeon IL-1β, E value < 1^e-30^) and PD002536 (chicken IL-1β, E value < 4^e-23^). These *in silico* analyses support that the sequence we cloned from the Chinese soft-shelled turtle is IL-1β.

Blast search reveals that the putative tcaspase-1 gene (GenBank Accession No. KM505034.1) is highly similar to chicken caspase-1 gene (GenBank Accession No. AF031351) (with 69% identity at the nucleotide level). The entire ORF is 1200 bp in length, encoding a 399 aa protein with a predicted molecular weight of 44.3 kDa. The SMART program analysis showed that the tcaspase-1 contained two domains, the CARD domain (1–89 aa, E value < 1.82^e-13^) and the CASc domain (146–397 aa, E value < 3.86^e-108^) as well as the conserved pentapeptide QACRG at aa 284–288 (Figure S1), which is considered as the active center [32].

### 3.2. The tIL-1β Gene Organization and Phylogenetic Tree

**Figure 3:**
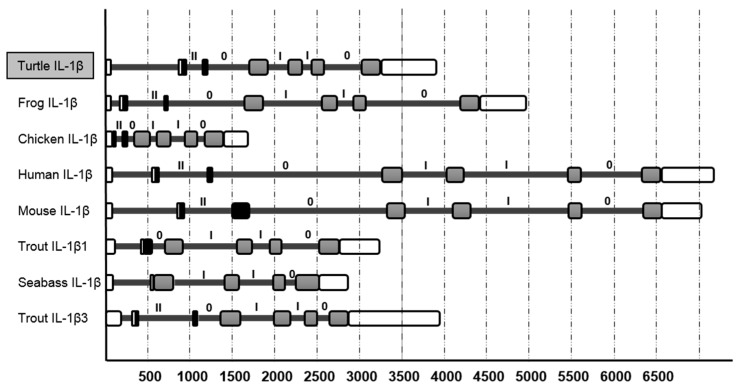
Comparison of the gene organizations and intron/exon sizes between *P. sinensis* IL-1β and known IL-1β genes. The coding regions are represented by filled boxes and the last four exons coding for the region of β-trefoil are filled in grey. The UTRs are depicted with an open box. Introns are shown as a black line with the intron phase above them. The accession numbers of the IL-1β sequences are as follows: AJ314758 (African clawed frog IL-1β), ENSGALT00000000738 (chicken IL-1β), ENST00000263341 (human IL-1β), ENSMUST00000028881 (mouse IL-1β), AJ278242 (trout IL-1β1), AJ311925 (seabass IL-1β), AM18168 (trout IL-1β3).

Analysis of tIL-1β with the SIM4 program reveals that it consists of seven exons and six introns, and has a similarly sized exon as those of IL-1β genes in mammals, birds, fish and amphibians (Figure 3). The exon of tIL-1β has the typical arrangement (II, 0, I, I, 0) within the putative coding region. Its twelve β-sheets structure peptide is located in the last three exons. This organization is similar to the known IL-1 family members except that some fish species lack the 5’ end intron (Figure 3).

Multiple alignment shows that tIL-1β possesses a relatively conserved region in the C-terminus forming the β-sheet that is considered a feature of IL-1 family (Figure 2). Human IL-1β contains the aspartic acid (Asp/D) 116, as the ICE cut site. The conserved aspartic acid residue could be found in IL-1β of mouse, rat and other mammals, but absent in IL-1β of turtle, fish, frog and chicken (Figure 2).

**Figure 4:**
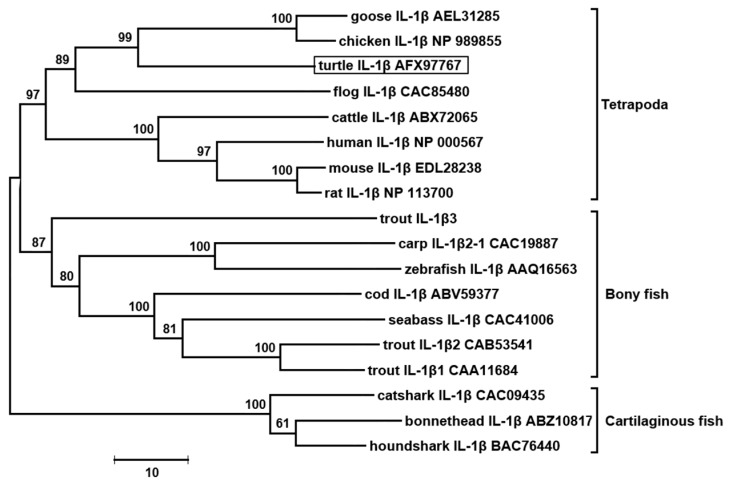
Unrooted phylogenetic tree showing the relationship between the amino acid sequence of *P. sinensis* IL-1β and other known IL-1β sequences. The tree is bootstrapped 10,000 times and the bootstrap values are indicated.

The phylogenetic NJ-tree of vertebrate IL-1β was constructed. As shown in Figure 4, tIL-1β together with IL-1β molecules from mammals, birds and amphibians fell into the same clade, and IL-1βs from fish form another clade. In the clade of tetrapod, tIL-1β clustered together with bird IL-1β with a high bootstrap confidence value (99%). The differences among the vertebrate IL-1βs reflect their phylogenetic distance.

### 3.3. Expression of tIL-1β in Different Tissues of Clinically Healthy Turtles or Aeromonas Hydrophila-Infected Turtles

**Figure 5:**
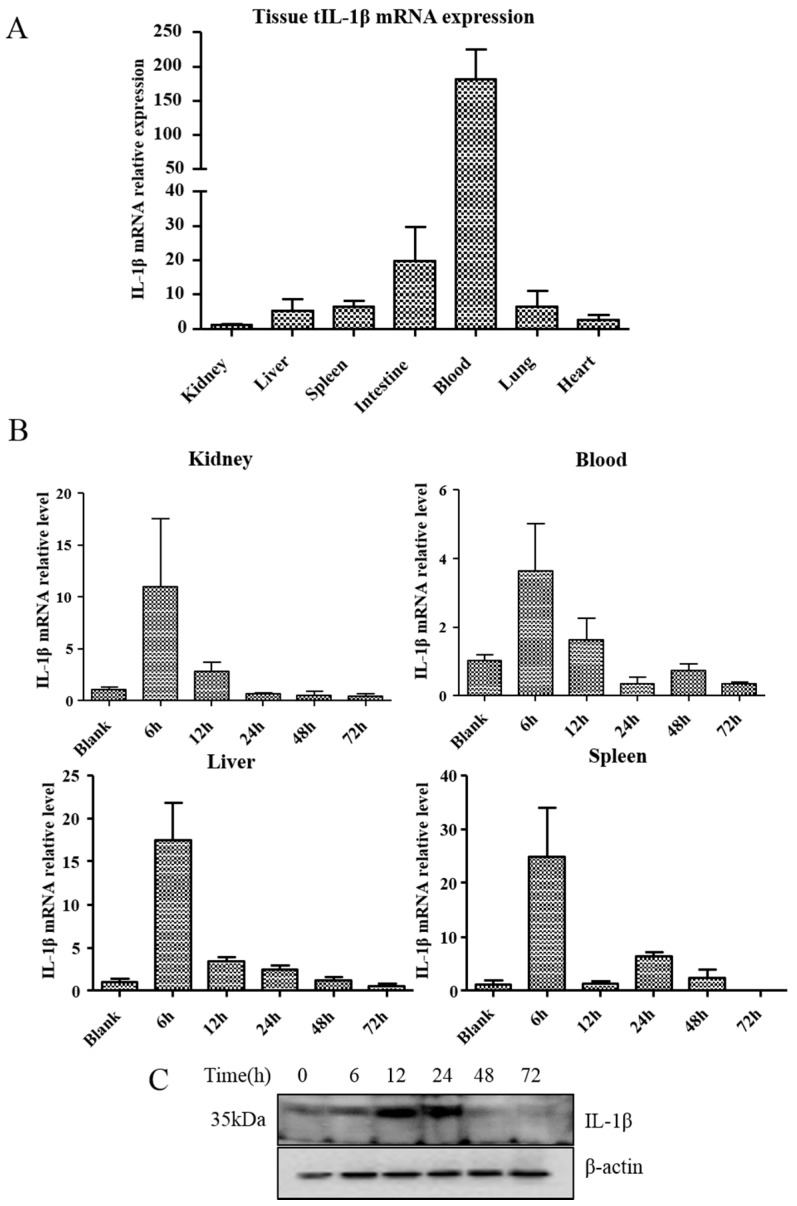
Analysis of the tissue expression of *P. sinensis* IL-1β in clinically healthy turtles (A) or *Aeromonas hydrophila*-infected turtles (B,C). (**A**) Quantitative PCR was performed using primers specific for tIL-1β with cDNA extracted from different tissues. β-actin was used as internal control and the results were normalized relative to the expression level in kidney. The results were shown as mean ± SEM of five turtles; (**B**) The mRNA levels relative to β-actin in kidney, blood, spleen and intestine from infected turtles were normalized with the mock group (*i.e.*, uninfected turtle tissue) set at 1.0; (**C**) Protein extracted from spleen samples of infected turtles were subjected to western blotting for tIL-1β and β-actin.

Expression profiles of tIL-1β mRNA in turtle tissues were measured by quantitative PCR. Figure 5A shows that in clinically healthy turtles, tIL-1β transcription was the highest in blood, followed by intestines and spleen. β-actin was used as internal control and the expression level in each organ was calculated relative to the kidney (having the lowest IL-1β transcripts) set at 1.0 for easy comparison [33]. In *A. hydrophila*-infected turtles, tIL-1β transcript expression peaked at 6 h post-infection (hpi) in spleen, followed by liver and kidney, and rapidly fell back to normal (Figure 5B). Western blotting shows that tIL-1β expression in the spleen was significantly elevated at 12 and 24 hpi, and declined markedly from 48 to 72 hpi compared to the level of uninfected turtles (Figure 5C).

### 3.4. Processing of tIL-1β by tcaspase-1 in HEK293 Cells

**Figure 6:**
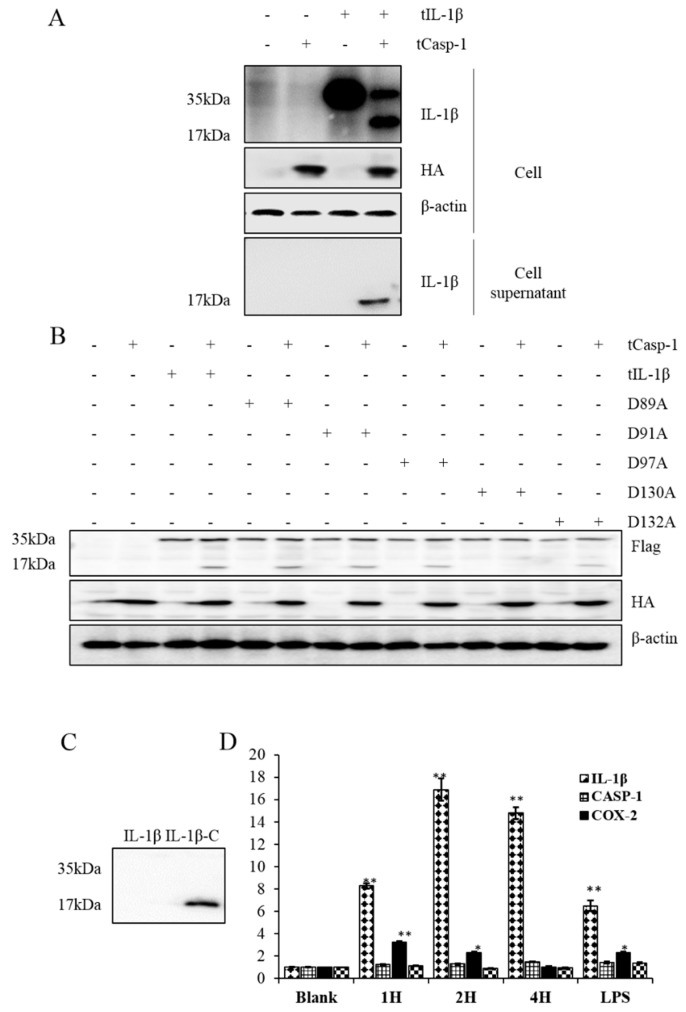
(**A**) The HEK293 cell was transfected with pcDNA-tcasp-1-HA and/or pcDNA-tIL-1β-Flag. Culture supernatant samples and cell lysates were collected 36 h post-transfection and detected by Western blotting; (**B**) HEK293 cells were co-transfected with wild-type pcDNA-tIL-1β-Flag/mutant plasmids and pcDNA-tcasp-1-HA. Whole-cell lysates were subjected to Western blotting for the target proteins; (**C**) Expression of truncated tIL-1β from D130 to C-terminus in Sf9 cells; (**D**) The mRNA transcripts from stimulated PBMo were normalized to the non-stimulated group and results are expressed as mean ± SEM of three independent experiments.

Western blotting was used to examine the cleavage of tIL-1β protein in HEK293 cells transfected with pcDNA-tcasp-1-HA and/or pcDNA-tIL-1β-Flag. Two different tIL-1β peptides about 35 kDa and 17 kDa (as the precursor and putative mature peptide of tIL-1β) could be detected in the cells co-transfected with both plasmids (Figure 6A). Supernatant samples were immune-precipitated with polyclonal antibody against tIL-1β to examine secreted tIL-1β in the culture medium. Figure 6A shows that the 17 kDa putative mature tIL-1β peptide was present only in the supernatant of co-transfected cells. No such tIL-1β peptide was detected in the cells expressing tIL-1β alone. These results reveal that tcaspase-1 was involved in the process of tIL-1β, and only the mature tIL-1β could be secreted into the culture medium.

Caspases cleave the substrates at aspartic acid residues [34]. Inspection of the tIL-1β protein sequence reveals that it contained a potential tcaspase-1 cleavage motif between aspartic acids 89 and 130. To identify the potential cleavage site, we mutated aspartic acid residues of predicted tIL-1β ICE cut site: D89, D91, D97 and D130 to alanine, respectively. As shown in Figure 6B, D130A mutation abolished tIL-1β cleavage by tcaspase-1. These results indicate that turtle caspase-1 could cleave turtle IL-1β at aspartic acid 130.

To address the function of tIL-1β peptide cleaved at D130, we tried to produce turtle IL-1β precursor and truncated turtle IL-1β (T131-278H) using the baculovirus expression vector system. Figure 6C shows that only the mature form of tIL-1β (tIL-1β-C), and not the full length of tIL-1β, was expressed. tIL-1β-C was used to stimulate turtle peripheral blood monocytes (PBMo) for transcriptional analysis of several inflammation-related molecules. LPS-treated cells were used as positive control. Stimulation of tIL-1β-C peptide resulted in increment of mRNA transcription of *tIL-1β* in PBMo at 4 h post-treatment (hpt) (Figure 6D). Transcription of *cox-2* was also increased at 2 hpt, but reduced to normal levels at 4 hpt. Mature tIL-1β peptide did not seem to have any effects on the transcription of *IL-18* or *casp-1.* These results suggest that the peptide cleaved at site D130 is bioactive.

## 4. Discussion

Interleukin-1β, an apical pro-inflammatory cytokine with multiple functions, has been extensively studied in species of mammals, birds, fish and amphibians, but not yet recognized in reptiles [2]. In the present study, the full-length cDNA of IL-1β from Chinese-soft shelled turtle was characterized. Typically, vertebrate IL-1β gene contains numerous copies of instability motif “ATTTA” in the 3’-UTR, which is typical of genes coding for inflammatory mediators and relating to the stability of mRNA in cytoplasm [35]. However, tIL-1β has only one copy of “ATTTA” instability motif in 3’-UTR. There is as of yet no study on turtle inflammatory mediators in regards to ATTTA motif. We analyzed some inflammatory mediators in *P. sinensis* available in the database, IL-8 (FJ472847) has nine ATTTA motifs, COX-2 (XM_006110862) has 30 ATTTA motifs and partial sequence of TNF-α (XM_014575959) has at least four; ATTTA motif copy numbers of these genes have no significant difference with their homologous counterparts in other vertebrates. GC content of tIL-1β coding sequence is 62%, similar to that of chicken (64%) and swan goose (67%), but significantly different from other vertebrates (42.0%–50.8%, for the sequence in Figure 4). Moreover, GC content in 3’-UTR of tIL-1β (65.6%) is remarkably higher than in any other vertebrate IL-1βs (28.5%~48.5%, for the sequence in Figure 4). High GC content in the coding sequence and 3’ UTR might account for increased molecular stability [36]. These evidences suggest tIL-1β mRNA has longer half-life in the cytoplasm, which is unusual since inflammatory IL-1β is tightly regulated at the mRNA level [37]. It indicates the difference between tIL-1β and other vertebrates IL-1β in regulation of mRNA. Whether it is a specific case or is common in reptile needs more investigation.

The tIL-1β clusters with IL-1β from avian and amphibian species. The IL-1β genes typically have seven exons and six introns with the last three intron phase as (1, 1, 0). Though there is close evolutionary relatedness [38] with high identity of IL-1β genes between turtle and chicken, their genetic organizations are different in that tIL-1β does not have the first non-coding exon. Notably, the intron-exon organization of the IL-1β genes of fish differs from other species (Figure 3). There are two types of IL-1β genes in fish as a result of genome duplication: one has the typical gene organization and the other does not have exons 1−3 which encode the C terminus β-trefoil part [39]. Husain suggested that the reduction in exon number is likely a fusion event of the exons encoding the N-terminus pro-domain of the protein [1]. Comparison of the intron size of different vertebrates reveals that Chinese soft-shelled turtle is similar to clawed frog and fish in intron size, but smaller than mammals. This might be the result of genomic duplication of IL-1 family genes during evolution [40].

In mammals, IL-1β is mainly expressed in macrophages and monocytes. However, the expression patterns of IL-1β vary with organs of the various organisms under normal conditions. Jiang, *et al.* showed that the expression of IL-1β mRNA is low in the kidney, spleen, gill and intestine, and absent in the liver, heart and muscle in sea bream [41,42,43]. The Atlantic bluefin tuna IL-1β has higher expression in liver than that in head kidney [44]. The transcription levels increased significantly in bacterial-infected salmonids and trout (50 folds and 1000 folds, respectively), depending on the stimuli used [1,45]. We found that tIL-1β mRNA is constitutively expressed at relatively high levels in peripheral blood, intestine, spleen, lung and liver (Figure 5A). It is also highly responsive upon *A. hydrophila* infection, its expression increased by 4- to 20-fold in examined tissues at 6 hpi, but declined rapidly from 12 hpi (Figure 5B). Dinarello indicated that transient transcription of IL-1 mRNA could be triggered and accumulated in response to stimuli in initial hours, but decreased rapidly due to the synthesis of a transcription repressor [46].

IL-1β precursor is not fully active until it is proteolytically cleaved and transported out of the cell. Caspase-1, whose activation is mediated by inflammasome, promotes secretion of the proinflammatory cytokines IL-1β [47]. By co-expressing of tIL-1β and tcaspase-1 in HEK293 cells, we found tcaspase-1 is able to process tIL-1β into peptide approximately 17 kDa; as predicted, processed peptide could be secreted, which is detectable in the supernatant (Figure 6A). The IL-1β from all mammalian species have a consensus ICE cut site at aspartic acid around aa 120, which is absent in other vertebrate species [48,49]. The tIL-1β, like other non-mammalian vertebrates, also lacks ICE cut site. A recent investigation has indicated that the cleavage site within all known IL-1β molecules is located at the 9 amino acids N-terminal to AXD, a conserved motif, where A is an aliphatic amino acid [50]. The motif is also present in the tIL-1β as 128IXD130, in consensus with other IL-1β sequences. This might be a cleavage site leading to a C-terminus peptide lacking the first β-sheet in non-mammalian vertebrate IL-1β. In our study, mutation of D130 to alanine acid prevents tcaspase-1 from processing the tIL-1β (Figure 6B), indicating D130 is the cleavage site of caspase-1 in tIL-1β. Vojtech, *et al.* demonstrated caspase A and caspase B from zebrafish are able to process IL-1β. Caspase A, orthologs of caspase-1, could cleave at D122 of zebrafish IL-1β, which is the consensus sequence of tIL-1β D130 (Figure 2) [49]. However, it is unknown whether the cleavage product of tIL-1β at D130 has bioactivity. We expressed the truncated tIL-1β peptide from D130 to C-terminus using the baculovirus expression system (Figure 6C) and found that the peptide has a similar ability to the mature IL-1β in enhancing the transcription of *IL-1β* itself and *cox-2* in PBMo (Figure 6D). This evidence supports that D130 is the ICE cut site in tIL-1β.

## 5. Conclusions

In summary, we have identified and characterized interleukin-1β (IL-1β) from Chinese soft-shelled turtle. It participates in immune response in bacterial infection. As other non-mammalian vertebrates, it lacks the typical IL-1β-converting enzyme (ICE) cut site, but could be cleaved at D130 to generate bioactive peptide. Our work will help further investigation of the immune and inflammatory responses of Chinese soft-shelled turtle to microbial infections and molecular evolution of IL-1 family cytokine.

## Supplementary Materials

The following are available online at www.mdpi.com/2073-4425/7/5/18/s1, Figure S1: Nucleotide and amino acid sequence of partial cDNA of *P. sinensis* caspase-1. The start and stop codons are boxed, CARD domain of caspase-1 is highlighted in grey and CASC domain is highlighted in black. The caspase-1 pentapeptide active-site motif sequence QACRG is highlighted in red., Table S1: PCR primers.
