# Supplementary material for: Identification and Functional Analysis of Interleukin-1β in the Chinese Soft-Shelled Turtle Pelodiscus sinensis
Source: Genes (Basel). 2016 May 4;7(5):18. doi: 10.3390/genes7050018 (PMC4880838; doi:10.3390/genes7050018)
Supplement: Supplementary file 1 [file genes-07-00018-s001.pdf]

```
1       tgcaggccgggccgggcgggccctgagcgATGGCGGATCGGCAGCTCGGCAGCGTGCGGACCAGGTTCGTGG
1                                    M  A  D  R  Q  L  G  S  V  R  T  R  F  V

73      AGAGCGTGAGCAAAGCCGTGATCAACTCCCTCCTGGACGACCTGCTGGAGAAGCAGGTGCTGAACGAGGAGG
15      E  S  V  S  K  A  V  I  N  S  L  L  D  D  L  L  E  K  Q  V  L  N  E  E

145     AGGTGGAGGAGGTGAGGGAAGGCCACAGCAAGAAGAGTGACCAAGCCAGGTGTCTGATCGATGGGGTGAGGA
39      E  V  E  E  V  R  E  G  H  S  K  K  S  D  Q  A  R  C  L  I  D  G  V  R

217     AAAAGGGAGCCAGAGCCAGCGAGATCTTCATCCGGTGCCTCTGCTGCAGAGACGTGCACCTGGCCAGTGACC
63      K  K  G  A  R  A  S  E  I  F  I  R  C  L  C  C  R  D  V  H  L  A  S  D

289     TGGGGCTGGCAGCCCCCTCGGCTACCGCAGGGGTTCCAGTGCCACAGCCAGGGCCGGCCCCCCAGGAGAACG
87      L  G  L  A  A  P  S  A  T  A  G  V  P  V  P  Q  P  G  P  A  P  Q  E  N

361     AGCCAGGGTCCACCGCAGAAACCCAGCCAGCGCCATGCGAGGGCTGGATCCGGCCCTGCCCCCCGGAGGACG
111     E  P  G  S  T  A  E  T  Q  P  A  P  C  E  G  W  I  R  P  C  P  P  E  D

433     TCCAGCGGATCCAGAGGGAGGAAGCAAAGGAGATCTATCCCATCCGGGACAAGGCAACACGAACCCGCCTGG
135     V  Q  R  I  Q  R  E  E  A  K  E  I  Y  P  I  R  D  K  A  T  R  T  R  L

505     CCCTCATCATCTGTAACGTAGAGTTCGAGCATCTCCCCAGGCGGGGCGGGGCCGACGTGGACGTGAGTGGGA
159     A  L  I  I  C  N  V  E  F  E  H  L  P  R  R  G  G  A  D  V  D  V  S  G

577     TGCAGAGGCTTCTAGAGGGGCTGGGCTACAAGGTGGAAACCTACTGCAACTTACCCGCCCAGGACATGCTGG
183     M  Q  R  L  L  E  G  L  G  Y  K  V  E  T  Y  C  N  L  P  A  Q  D  M  L

649     CAACGCTGAAGCAGTTTGCTGCTCGGGACGAGCACCAGACCTCGGACAGCACCTTCCTTGTGCTCATGTCCC
207     A  T  L  K  Q  F  A  A  R  D  E  H  Q  T  S  D  S  T  F  L  V  L  M  S

721     ATGGCGTCCGGGCAGGGCTGTGCGGGACGAAGAGCCACGGCGGGGCCACGGACATCCTTCCCGTCGACACCA
231     H  G  V  R  A  G  L  C  G  T  K  S  H  G  G  A  T  D  I  L  P  V  D  T

793     TCTACAGCACCTTCAACAACAAGAGCTGCCAGGCCCTGCTGGGCAAGCCCAAAGTGATCATCATCCAGGCCT
255     I  Y  S  T  F  N  N  K  S  C  Q  A  L  L  G  K  P  K  V  I  I  I  Q  A

865     GCCGGGGGGAGAGCCAGGGACACGTGTGGGTGAGCGACTCCGCAGAGCTCCCTGGAGATGGCGCCAGCCCTG
279     C  R  G  E  S  Q  G  H  V  W  V  S  D  S  A  E  L  P  G  D  G  A  S  P

937     CGCCATGGCCCACCGAAGAGCTGGAAGATGATGCCACCCACCAAATCCACGTGGAGAGCGATTTCATCTGCT
303     A  P  W  P  T  E  E  L  E  D  D  A  T  H  Q  I  H  V  E  S  D  F  I  C

1009    TCCACTCCACAACGCCAGACACCGTGTCCTGGAGAAGTCCGAAAACTGGCTCCGTCTTCATCAAATGCCTGA
327     F  H  S  T  T  P  D  T  V  S  W  R  S  P  K  T  G  S  V  F  I  K  C  L

1081    TAGAGCAGCTCCAAACCAACGCCTGGCGCTTCCCCTTGGAGGAGATCTTCCGAAAGGTCCAGCTCTCCTTTC
351     I  E  Q  L  Q  T  N  A  W  R  F  P  L  E  E  I  F  R  K  V  Q  L  S  F

1153    AAAATTTTCCTCGTCAGATGCCCACAAAAGAGAGAACTACCATGCTGAAAAAGTTCTACCTGTTCCCCGGCC
375     Q  N  F  P  R  Q  M  P  T  K  E  R  T  T  M  L  K  K  F  Y  L  F  P  G

1225    ATTAGgacaagcccacgatatggcagagcatttcccgggggcccagcctcagtcagca
399     H  *
```

Figure S1. Nucleotide and amino acid sequence of partial cDNA of P. sinensis caspase-1. The start and stop codons are boxed, CARD domain of caspase-1 is highlighted in grey and CASCs domain is highlighted in black. The caspase-1 pentapeptide active-site motif sequence QACRG is highlighted in red.

Table S1. PCR primers.

Primers	Nucleotide sequence(5'–3')	Application(s)
tIL-1β-deg-F1	CAATCTCTACATGTCYTG	Amplifying intermediate fragments
tIL-1β-deg-F1	GGGGAAACCGGCMS	
tIL-1-R1	CGAACTGGGTGGTGTCCCCGCTCT	5′Race
tIL-1-R2	GCATGGGCTGCTGGCCGTTGTG	
GeneRacer 5′Primer	CGACTGGAGCACGAGGACACTGA	
GeneRacer 5′ nested Primer	GGACACTGACATGGACTGAAGGAGTA	
tIL-1-F1	CAGAGGCCCCGAGCTGGAGCGTT	3′Race
tIL-1-F2	GAGCGGGGACACCACCCAGTTCGA	
GeneRacer 3′Primer	GCTGTCAACGATACGCTACGTAACG	
GeneRacer 3′ nested Primer	CGCTACGTAACGGCATGACAGTG	
tcas-1-cF	CCTGAGCGATGGCGGATCGGCA	Tcaspase-1 cloning
tcas -1-cR	CGTGGGCTTGTCCTAATGGCCGG	
tIL-1-cF	CACATATGGCAGCTGTCCCCGACAT	t-IL-1beta precursor cloning
tIL-1-cR	TATCTCGAGGTGCTTGGCCTGCGTGAGCA	
tIL-1-g1F	GGGCCAGCACTCGCATCACA	Gene organization
tIL-1-g1R	CCGGGGGCTCAGTGCTTGGC	
tIL-1-g2F	ATCACAGGTTTCACCACCAGACGCAGCCAT	
tIL-1-g2R	TGCGTGAGCAGGTAGTTGGTGATGAACACC	
tIL-1-Flag-F	CTCGGATCCCAGGTTTCACCACCAGACGCAGCC	pcDNA3-tIL-1β-Flag
tIL-1-Flag-R	TATCTCGAGGTGCTTGGCCTGCGTGAGCAGGTAG	
tCasp1-HA-F	ATTAAAGCTTGCCACCATGTACCCATACGATGTTCCAGATTACG<br>CTGCGGATCGGCAGCTCGGC	pcDNA3-tcasp1-HA
tCasp1-HA-R	TAATCTCGAGCTAATGGCCGGGGAACAGG	
rtIL-1-F	AATCATATGTACCGCTACCTCCGCTCCAT	Cloning of tIL-1β (Y118-276H) C terminal into pET32a
rtIL-1-R	TATCTCGAGGTGCTTGGCCTGCGTGAGCA	
tIL-1β-Bac-F	ACATTTCTTACATCTATGCGATGGCAGCTGTCCCCGACATGC	Cloning of tIL-1β into pFastBacHT-melittin
tIL-1β-Bac-R	CTCTAGTACTTCTCGACAAGCTTTAATGGTGATGGTGATGGTGA<br>TGGTGCTTGGCCTGCGTGAGC	
tIL-1β-Bac-131-F	ACATTTCTTACATCTATGCGATGACTGACCAGAAGTGCTTCAAG	Cloning of tIL-1β (131–277) into pFastBacHT-melittin
tIL-1β-Bac-131-R	CTCTAGTACTTCTCGACAAGCTTTAATGGTGATGGTGATGGTGA<br>TGGTGCTTGGCCTGCGTGAGC	
tIL-1-qF	CTTCTCGGACAATGACTT	IL-1β Q-RT-PCR
tIL-1-qR	GTAGCGGTAGATGGAATT	
COX-2-qF	TTGACCGATATGAATGTG	COX-2 Q-RT-PCR
COX-2-qR	AATGAGTGAGGATGTAGT	
IL-18-qF	GGTAGACAACAAGACTTAC	IL-18 Q-RT-PCR
IL-18-qR	CTCGCATTAGTGAAGATT	
tCasp-1-qF	ATCTTCCGAAAGGTCCAG	tCASP-1 Q-RT-PCR
tCasp-1-qR	TTCAGCATGGTAGTTCTCT	
β-actin-qF	GAGACCCGACAGACTACCT	β-actin Q-RT-PCR
β-actin-qR	AGGATGATGAAGCAGCAGT	
